# Cold weather is a predisposing factor for testicular torsion in a tropical country. A retrospective study: Clima frio é um fator predisponente para torção testicular num país tropical. Um estudo retrospectivo

**DOI:** 10.1590/1516-3180.2013.7600007

**Published:** 2014-09-26

**Authors:** Daniel de Oliveira Gomes, Rafael Rocha Vidal, Bruno Figueiredo Foeppel, Danilo Fiorindo Faria, Minori Saito

**Affiliations:** I MD. Attending Physician, Department of Urology, Hospital Santa Marcelina, São Paulo, Brazil.; II MD. Senior Resident, Department of Urology, Hospital Santa Marcelina, São Paulo, Brazil.; III MD. Attending Physician, Department of Pediatric Surgery, Hospital Santa Marcelina, São Paulo, Brazil.; IV MD. Department Head, Department of Urology, Hospital Santa Marcelina, São Paulo, Brazil.

**Keywords:** Spermatic cord torsion, Incidence, Cold climate, Seasons, Brazil, Torção do cordão espermático, Incidência, Clima frio, Estações do ano, Brasil

## Abstract

**CONTEXT AND OBJECTIVE:**

Testicular torsion is a medical and urological emergency because it can lead to loss of the organ. The theory of seasonal testicular torsion occurrence is based on studies from institutions located in cold and temperate regions. The objective here was to determine whether cold weather is associated with higher incidence of testicular torsion in a tropical country, such as Brazil.

**DESIGN AND SETTING:**

Retrospective study, conducted in a tertiary and teaching hospital.

**METHODS:**

Patients with acute testicular torsion confirmed by surgery between April 2006 and March 2011 were studied. Information on weather conditions at the time of symptom onset was collected.

**RESULTS:**

A total of 64 testicular torsion cases were identified. The months with the highest incidences of testicular torsion were June (16%), July (19%) and August (11%), which had the lowest mean temperatures, of 17.6 °C, 16.4 °C and 18.2 °C, respectively. Eleven percent of cases occurred during spring (October to December), 16% occurred in summer (January to March), 34% occurred in fall (April to June) and 39% occurred in winter (July to September). There was a significant association between the incidence of testicular torsion and the season (fall and winter), P < 0.001.

**CONCLUSIONS:**

Testicular torsion follows a seasonal association even in a tropical country, and is more frequent in the colder months of the year, namely fall and winter, when almost three-quarters of the cases occurred. These observations add further evidence that cold weather has an etiologic role in testicular torsion occurrence.

## RESUMO

**CONTEXTO E OBJETIVO:**

A torção testicular é uma emergência médica e urológica, pois pode levar à perda do órgão. A teoria da ocorrência sazonal da torção testicular é baseada em estudos de instituições localizadas em regiões frias e temperadas. O objetivo foi determinar se o clima frio está associado a uma maior incidência de torção testicular em um país tropical, como o Brasil.

**TIPO DE ESTUDO E LOCAL:**

Estudo retrospectivo, realizado em um hospital terciário e de ensino.

**MÉTODOS:**

Foram estudados pacientes com torção testicular aguda confirmada na cirurgia, durante o período de abril de 2006 a março de 2011. Informações sobre as condições climáticas no início dos sintomas foram coletadas.

**RESULTADOS:**

Um total de 64 casos de torção testicular foi identificado. Os meses com maiores incidências foram junho (16%), julho (19%) e agosto (11%), que assinalaram as menores temperaturas médias, de 17,6 °C, 16,4 °C e 18,2 °C, respectivamente. Onze por cento dos casos ocorreram durante a primavera (outubro a dezembro), 16% no verão (janeiro a março), 34% no outono (abril a junho) e 39% no inverno (julho, agosto, setembro). Houve associação significante entre a incidência de torção testicular e a estação (outono e inverno), P < 0,001.

**CONCLUSÃO:**

Torção testicular obedece a uma predileção sazonal mesmo num país tropical, sendo mais frequente nos meses mais frios do ano, particularmente no outono e inverno, quando ocorrem quase três quartos dos casos. Estes achados acrescentam evidência a um papel etiológico do clima frio na torção testicular.

## INTRODUCTION

Testicular torsion is a medical and urological emergency, because it can lead to irreversible ischemic damage and loss of the organ. The annual incidence of this disorder in men younger than 25 years of age is 1:4000.^1^ Approximately 61% of patients with testicular torsion are under 21 years of age, with a bimodal age distribution of two peaks: one in the neonatal period and the other at 13 years of age.^2^ Testicular torsion usually occurs in the absence of precipitating factors,^3^ and only 4% to 8% of the cases result from trauma.^4^ Other factors that enhance patients’ risk of testicular torsion are increased testicular volume (usually associated with puberty), history of cryptorchidism, presence of testicular tumors and testes with a greater horizontal axis and with a long intrascrotal spermatic cord.^2^^,^^5^ It has been speculated that the hyperactive cremasteric reflex in the presence of cold weather represents another mechanism for testicular torsion. The theory of seasonal testicular torsion occurrence is based on studies from institutions located in cold and temperate regions.^6^^,^^7^^,^^8^^,^^9^^,^^10^^,^^11^^,^^12^ To our knowledge, no study in a tropical country has tested this hypothesis among patients with a diagnosis of testicular torsion confirmed by surgical examination.

## OBJECTIVE

We sought to determine whether lower atmospheric temperatures in a tropical country, such as Brazil, would be associated with higher incidence of testicular torsion.

## METHODS

### Study design

We performed a retrospective analysis on all patients with acute testicular torsion seen at a tertiary care hospital in São Paulo, Brazil, between April 2006 and March 2011. All the clinical data were obtained from the patients’ medical records. This study was approved by the Ethics Committee of our institution.

### Subjects and clinical data

Only the patients with a diagnosis of testicular torsion confirmed by surgical examination were included. Any patients with neonatal torsion and torsion of the appendix testis were excluded from the study. The date of symptom onset was recorded and tabulated with regard to the month and season of the year. In Brazil, fall occurs in April, May and June, and winter occurs in July, August and September. The mean and minimum atmospheric temperature of the month corresponding to the date of symptom onset for each patient were obtained from the National Meteorological Institute, based on data recorded at the Meteorological Station of São Paulo.

### Statistical analysis

Statistical analysis was performed using the Statistical Package for the Social Sciences (SPSS 16.0) (IBM, Chicago, IL, USA) and the groups were compared using the chi-square test. Statistical significance was set at P < 0.05.

## RESULTS

**Figure 1.**
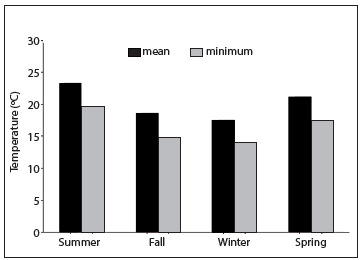
Minimum and mean temperatures during different seasons in São Paulo between 2006 and 2011.

A total of 64 cases of testicular torsion met the study inclusion criteria and there were no sample losses. The mean patient age was 16 years (median age, 17 years; range, 1 to 30 years) and the mean ambient temperature during the 60 months studied was 21 °C (range: 16 °C to 25 °C). The months with the highest incidence of testicular torsion were June (16%; 10/64), July (19%; 12/64) and August (11%; 7/64), which had the lowest mean temperatures of 17.6 °C, 16.4 °C and 18.2 °C, respectively, as well as the lowest minimum temperatures of 13.3 °C, 12.3 °C and 13.7 °C, respectively. Fifty-three of the 64 cases (83%) occurred when the minimum temperature was below 17.3 ºC, whereas only 11 patients had testicular torsion at minimum atmospheric temperatures above 17.3 °C (P < 0.001). Eleven percent of the cases (7/64) occurred during the spring (October, November and December), 16% (10/64) occurred during the summer (January, February and March), 34% (22/64) occurred during the fall (April, May and June) and 39% (25/64) occurred during the winter (July, August and September). Figure 1 shows the minimum and mean temperatures in different seasons in São Paulo between 2006 and 2011.

**Figure 2.**
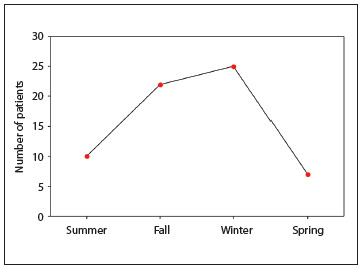
Seasonal frequency of acute testicular torsion.

Significant differences in the frequency of testicular torsion were observed between summer and fall (P = 0.012), summer and winter (P = 0.002), fall and spring (P = 0.001) and winter and spring (P < 0.001). As expected, there was no significant difference in testicular torsion frequency between fall and winter (P = 0.582) or between spring and summer (P = 0.434). Figure 2 shows the seasonal frequency of acute testicular torsion. Therefore, 73% (47/64) of the testicular torsion cases occurred during cold weather (fall and winter) and 27% (17/64) occurred during warm weather (spring and summer), P < 0.001.

## DISCUSSION

In this study, we identified higher incidence of testicular torsion in the colder months of the year, particularly during the fall and winter, even in a tropical region with an average temperature of 21 °C over the last five years. An atmospheric temperature drop to 17 °C or less is a risk factor for the occurrence of testicular torsion in Brazil. These findings, together with those from other authors, suggest that cold can cause contraction of the cremaster muscle and/or the tunica dartos and produce testicular torsion.^6^^,^^7^^,^^10^^,^^11^^,^^12^

Moreover, for most of our patients, symptom onset occurred during the night (data not shown), which may be related both to the temperature decrease, when the minimum temperatures are reached, and to the presence of “erotic dreams” during sleep. Previous studies regarding the seasonality of testicular torsion have reported conflicting data. In an English study, in 1976, Williamson^6^ evaluated 275 children with confirmed testicular torsion and noted a statistically significant increase in its occurrence from November to February (fall/winter in that country). They were the first authors to demonstrate a possible association between cold weather and cremasteric hyperreactivity leading to testicular torsion.

Six years later, in a study in Ireland, Shukla et al.^7^ reported that 40 out of 46 cases of testicular torsion occurred when the ambient temperature was below 2 °C. Subsequently, other studies were published, with five of them demonstrating a positive association between low ambient temperatures and testicular torsion.^8^^,^^9^^,^^10^^,^^11^^,^^12^

However, all of these studies were conducted in cold and temperate regions, such as England, New York/United States, Scotland, Greece and Japan. Five other studies showed no association between cold weather and testicular torsion. Mabogunje.^13^ reviewed 131 confirmed cases of testicular torsion and, interestingly, did not observe a significant difference in the incidence of testicular torsion between the warm and cold months but found a significant difference in its incidence between low and high relative humidity.

Driscoll et al.,^14^ in Scotland, and other authors in Canada and in the United States did not find any seasonal variation in the occurrence of testicular torsion.^15^^,^^16^^,^^17^ However, Cost et al.^17^ studied patients from regions all across the United States, a country with very different climatic regions, and Preshaw^15^ conducted a study in Calgary, Canada, where there is a long cold season, with extreme daily temperature variations and little monthly temperature variation. In these scenarios, finding higher incidence of testicular torsion in the months corresponding to fall/winter would be very difficult. Some of these authors who have not reported findings demonstrating testicular torsion occurrence with seasonal predilection have argued that there is a marked difference between indoor and outdoor temperature, since in most cases the onset of testicular torsion symptoms occurred indoors.^14^^,^^15^

These reports are from cold countries, where home heaters are quite commonly used, and such use increases the indoor/outdoor temperature difference. In our region, the ambient temperatures vary but within a comfortable range, and thus heaters are rarely used. The other strengths of our study are that it included only patients with testicular torsion confirmed by surgical examination and that it used data on atmospheric temperatures from the meteorological station closest to the area where our patients lived, and not “historical data” as in some of the abovementioned studies. A study recently published in Brazil reported an increase in the occurrence of torsion during the winter,^18^ but it included patients with testicular torsion presumed on admission, thus making it difficult to establish precise conclusions, because only 16% to 42% of boys with acute scrotal pain have surgically confirmed testicular torsion.^19^^,^^20^^,^^21^ Hypothesizing that cold weather is the main initiating event for acute testicular torsion would be too simplistic.

Although anatomical abnormalities are quite common in patients with testicular torsion,^22^ such as testicles with a greater horizontal axis and a spermatic cord with a long intrascrotal portion, only a small proportion of patients with anatomical abnormalities have testicular torsion.^23^ Cold weather and the consequent cremasteric hyperreactivity could be predisposing factors for torsion in individuals with unfavorable anatomy. The pathophysiology of testicular torsion is certainly more complex than postulated today, and there may be ultrastructural differences in the cremaster muscle tissue among men with and without torsion that have not yet been identified.

Our study has the limitations inherent to any retrospective study. Moreover, even in prospective studies, it would be difficult to correlate low ambient temperatures with torsion, given that the onset of the patients’ symptoms may be several hours before presentation at a hospital. Thus, obtaining the correct ambient temperature at the exact time of onset would be both complex and unrealistic.

Our findings corroborate the information from the literature regarding the role of cold weather on occurences of testicular torsion. Lower ambient temperatures, even in a tropical region, with pleasant atmospheric temperatures, might predispose individuals to torsion. This information could be used to recommend the use of warmer and more protective clothing for the external genitalia, for young boys during cold periods.

## CONCLUSIONS

Testicular torsion follows seasonal predilection even in a tropical country, and is more frequent in the colder months of the year, i.e. fall and winter. Nearly three-quarters of the cases occurred during this period. In our region, 83% of the testicular torsion cases occurred when the atmospheric temperature was 17 °C or less. Taken together, these findings support the hypothesis that cold weather has an etiological role in testicular torsion and provide evidence that this association is not coincidental.
